# SKILLS AND STRATEGIES OF FACILITATORS WHO CO-CREATE DIGITAL STORIES WITH PERSONS WITH DEMENTIA

**DOI:** 10.1093/geroni/igz038.1697

**Published:** 2019-11-08

**Authors:** Christine H Daum, Lili Liu, Kara Hollinda, David Kaufman, Arlene Astell

**Affiliations:** 1 University of Alberta, Edmonton, Alberta, Canada; 2 Independent, Saskatoon, Saskatchewan, Canada; 3 Simon Fraser University, Surrey, British Columbia, Canada; 4 University of Toronto, Toronto, Ontario, Canada

## Abstract

Digital storytelling combines storytelling and digital tools to create brief video clips in which narrative, images, and music are embedded, in order to share personal stories. Digital storytelling facilitators can be health and social care providers as well as care partners who collaborate with persons living with dementia to co-create their stories. These facilitators elicit people’s stories and use the technology to create the digital story. Despite their important role, there is a paucity of information on facilitators’ specific skills and strategies used in working with persons living with dementia. The purpose of this project was to examine skills and strategies used by facilitators who co-create digital stories with persons living with mild dementia. Audio recordings of 70 digital storytelling co-creation sessions conducted in three Canadian cities (Edmonton, Vancouver, Toronto) were transcribed and subjected to qualitative content analysis. Regardless of their disciplinary background, facilitators acted as weavers, bringing together narrative threads to co-construct a digital story with participants. Essential communication skills and strategies included active listening, strategic questioning, being comfortable with silence, and therapeutic responding. Building relationships and collaboration were achieved through flexibility, empathy, and encouraging autonomy. To be an effective facilitator of the digital storytelling process with older adults living with dementia, facilitators must adapt their communication strategies and relational skills to the strengths and needs of the older adults with whom they are collaborating.

